# STK19: a critical factor coordinating transcription-coupled DNA repair

**DOI:** 10.1093/procel/pwaf007

**Published:** 2025-01-31

**Authors:** Shisheng Li, Wentao Li

**Affiliations:** Department of Comparative Biomedical Sciences, School of Veterinary Medicine, Louisiana State University, Baton Rouge, LA 70803, United States; Department of Environmental Health Science, College of Public Health, University of Georgia, Athens, GA 30602, United States

Transcription-coupled DNA repair (TCR) is a subpathway of nucleotide excision repair (NER) that preferentially removes transcription-blocking DNA damage from the transcribed strand of active genes (Selby et al., 2023; van den Heuvel et al., 2021). Defects in TCR are implicated in severe human diseases, including Cockayne syndrome and UV-sensitive syndrome. Although TCR was discovered nearly 40 years ago and multiple proteins have been identified to be involved in this process (Li and Li, 2017), key questions about its molecular mechanisms remain unresolved. Unlike global genome repair (GGR), the other NER subpathway, which has been successfully reconstituted *in vitro*, TCR has been proven particularly challenging to reconstitute, likely due to its reliance on sequential and dynamic protein interactions, as well as the potential involvement of as-yet-unidentified factors in the repair process.

The TCR process is initiated when elongating RNA polymerase II (RNAPII) stalls at sites of DNA damage on the transcribed strand. This stalling event is the key signal that triggers TCR and sets off a coordinated series of protein interactions (Li et al., 2014; Selby et al., 2023; van den Heuvel et al., 2021; Xu et al., 2017). Cockayne syndrome protein B (CSB) senses the stalled RNAPII by binding to the upstream DNA and using its 3′ → 5′ translocase activity to push RNAPII forward (Kokic et al., 2021; Xu et al., 2017). This action displaces the elongation factor DSIF (Kokic et al., 2021; Selvam et al., 2019; Xu et al., 2017) and promotes the recruitment of the cullin-dependent CRL4^CSA^ ubiquitin ligase complex and UV-stimulated scaffold protein A (UVSSA) (van der Weegen et al., 2020). The transcription elongation factor ELOF1 positions CRL4^CSA^ and UVSSA in a way that promotes ubiquitination of K1268 in RPB1, the largest RNAPII subunit, and facilitates the recruitment of the transcription factor II H (TFIIH) complex (Geijer et al., 2021; Kokic et al., 2024; van der Weegen et al., 2021). The central unstructured segment of UVSSA interacts with the N-terminal pleckstrin homology domain of p62, a TFIIH subunit, which may also play a role in the initial recruitment of TFIIH (Okuda et al., 2017). The translocase and helicase activities, respectively conferred by XPB and XPD within the TFIIH complex, unwind the DNA and kinetically proofread the presence of damage, enabling the structure-specific nucleases XPF-ERCC1 and XPG to access and incise the damaged strand. Despite these advances, the molecular mechanisms underlying the precise recruitment and positioning of TFIIH within the TCR complex remain poorly understood, representing a critical gap in the field.

Four recent landmark studies have identified serine/threonine kinase 19 (STK19) as a key coordinator of TCR, using complementary approaches to reveal its structural interactions with other TCR proteins and implicate its role in positioning TFIIH within the TCR complex (Mevissen et al., 2024; Ramadhin et al., 2024; Tan et al., 2024; van den Heuvel et al., 2024). Initially annotated as a kinase, STK19 was later found to be a 254-amino acid DNA/RNA-binding protein containing three winged-helix domains but lacking kinase activity (Li et al., 2024; Rodriguez-Martinez et al., 2020). Early genome-wide screens and proteomic studies have suggested STK19’s involvement in the DNA damage repair and transcription recovery following DNA damage (Boeing et al., 2016; Olivieri et al., 2020). The absence of kinase activity raised questions about the mechanisms by which STK19 contributes to DNA repair and transcription recovery.

Tan et al. demonstrated that STK19 is essential for TCR using biochemical assays and next-generation sequencing-based methods, including XR-seq and Damage-seq (Tan et al., 2024). They revealed that STK19 is recruited to DNA damage sites following the recruitment of CSA during TCR. Notably, STK19 was shown to promote the UV-induced ubiquitination of UVSSA, but not RPB1, and facilitates the subsequent recruitment of TFIIH. Using *in vitro* pull-down assays, they confirmed that STK19 physically interacts with CSA, RNAPII, and TFIIH. Furthermore, AlphaFold2 predicted a direct interaction between STK19 and XPD, a subunit of TFIIH. They superimposed the TFIIH-XPA-DNA complex onto the RNAPII-TCR complex using the AlphaFold2-predicted CSA-STK19-XPD ternary complex, demonstrating that TFIIH can be loaded onto DNA downstream of RNAPII in the presence of STK19. This ground-breaking study provided compelling evidence for STK19’s critical role in linking CSA, RNAPII, and TFIIH recruitment during the TCR process.

Taking advantage of the low or undetectable endogenous levels of TCR factors in Xenopus egg extract, Mevissen et al. successfully reconstituted the TCR reaction by inhibiting XPC (the GGR-specific factor) and supplementing the extract with recombinant STK19, CSB, CRL4^CSA^, ELOF1, and UVSSA (Mevissen et al., 2024). This elegant approach demonstrated the requirements of canonical TCR factors and STK19 for the cell-free TCR reaction. The authors further resolved a high-resolution (1.9 Å) cryo-EM structure of the TCR complex, comprising *S. scrofa* RNAPII and human TCR factors STK19, CSB, CSA-DDB1, DDA1, ELOF1, and UVSSA. The structure revealed that STK19 integrates into the complex through interactions with CSA, RPB1, DDB1, UVSSA, and the DNA downstream of RNAPII. While the interaction with UVSSA was found to be nonessential for TCR, the interactions with CSA, RPB1, and DDB1 were demonstrated to be critical for the repair. The authors also present evidence that STK19 accommodates the downstream DNA but does not need to actively attract it to facilitate TCR. By combining their structural data with AlphaFold2-predicted interactions between STK19 and the XPD subunit of TFIIH, the authors generated a comprehensive model showing STK19 positioning TFIIH in front of the TCR complex adjacent to the downstream DNA. The functional significance of this arrangement was validated through mutational analysis of the STK19-XPD interface, establishing STK19’s role in coupling RNAPII stalling to downstream TCR events.

Van den Heuvel et al. demonstrated that STK19 associates with the RNAPII-bound TCR complex and is required for TCR (van den Heuvel et al., 2024). The loss of STK19 was found to delay the clearance of lesion-stalled RNAPII, with no impact on the initial assembly of the TCR complex or RPB1 ubiquitination, and only mild effects on UVSSA ubiquitination and the initial recruitment of TFIIH. The authors resolved a 3.3 Å cryo-EM structure of the TCR complex, comprised of *S. scrofa* RNAPII and human TCR factors STK19, CSB, CSA-DDB1, UVSSA, and ELOF1. The structure revealed that STK19 interacts with CSA, RPB1, UVSSA, and the DNA downstream of RNAPII. Mutations that attenuate any of the interactions were demonstrated to compromise TCR. AlphaFold prediction indicated that STK19 interacts with XPD. The STK19-XPD model could be superimposed with the structure of the TFIIH core complex onto the RNAPII-bound TCR complex without significant clashes, supporting a model where STK19 positions and stabilizes TFIIH in the RNAPII-bound TCR complex in an optimal configuration for XPD to start scanning the transcribed strand for the DNA lesion (Kim et al., 2023; van den Heuvel et al., 2024). The authors proposed that STK19 serves as the bridge between the initial TCR complex formation and downstream repair by enabling efficient TFIIH-mediated remodeling and ultimately displacement of RNAPII.

Ramadhin et al. demonstrated that STK19 is an integral part of the TCR complex, where it stimulates UV-induced ubiquitination of RPB1 and stabilizes the binding of UVSSA and TFIIH to the complex (Ramadhin et al., 2024). They resolved the cryo-EM structure of the TCR complex containing *S. scrofa* RNAPII and human TCR factors STK19, ELOF1, CSB, UVSSA, and the entire neddylated CRL4^CSA^ ubiquitin ligase complex. This structure revealed that STK19 interacts CSA, UVSSA, RPB1, and downstream DNA, consistent with observations from the other resolved TCR complexes (Mevissen et al., 2024; van den Heuvel et al., 2024). However, STK19 adopts multiple conformational states within the complex and may not engage all interaction partners simultaneously, potentially allowing it to coordinate with other factors. The neddylated CUL4A-RBX1, the catalytic module of the CRL4^CSA^ ubiquitin ligase complex, was found to be mobile, potentially enabling the ligase to access different targets within the TCR complex. While the structure does not indicate a direct interaction between STK19 and CUL4A-RBX1, STK19 was shown to stabilize the association of CUL4A-RBX1 with the TCR complex, thereby facilitating the ubiquitylation of RPB1, UVSSA, and CSB. Furthermore, superposition of the AlphaFold-predicted STK19-XPD model with a previously resolved TFIIH-XPA-DNA structure revealed a positively charged area of STK19 positioned adjacent to the 3′ single-stranded DNA exiting XPD. This suggests a critical role for STK19 in positioning TFIIH within the TCR complex.

**Figure 1.**
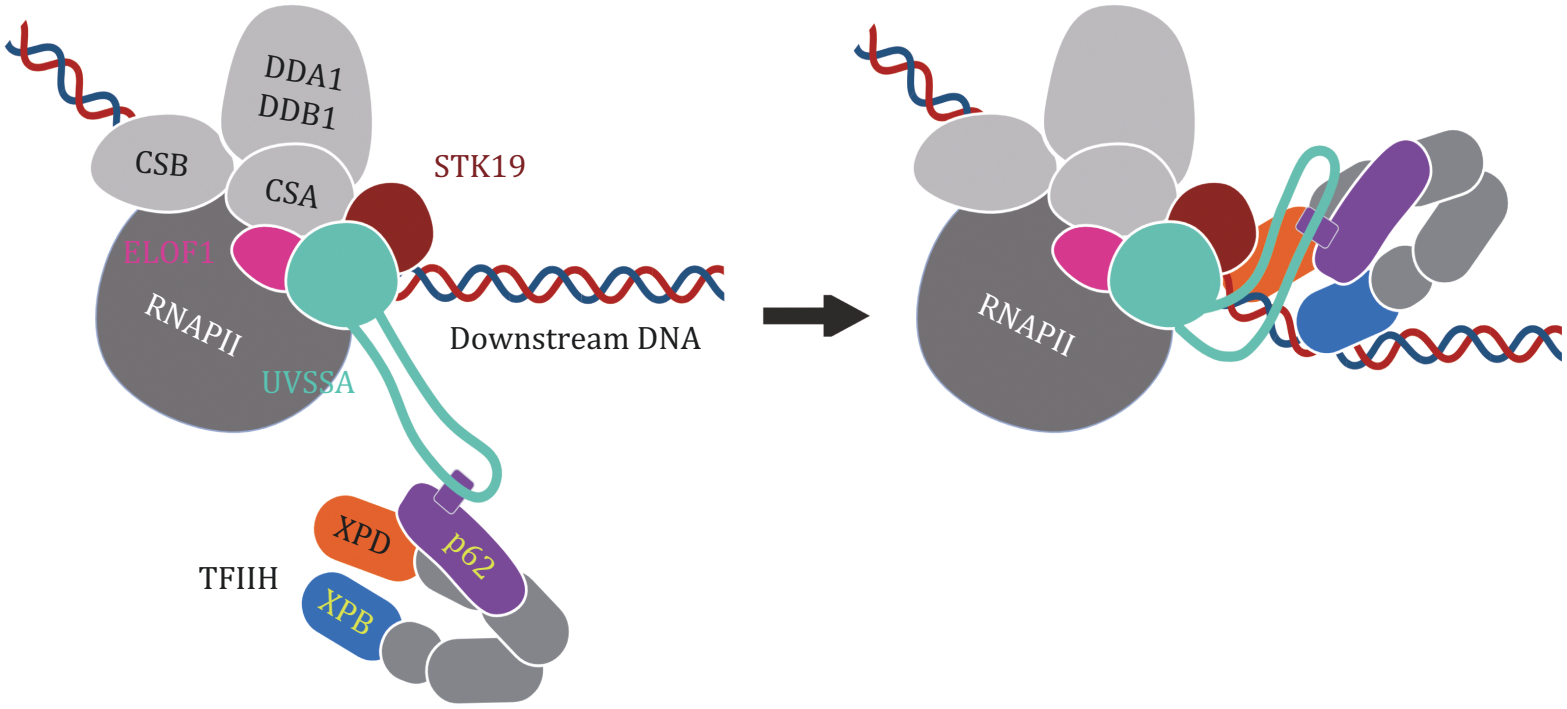
**Schematic representation of TCR initiation triggered by RNAPII stalling at sites of DNA damage.** The stalling event leads to the coordinated recruitment of CSB, the CRL4^CSA^ ubiquitin ligase complex (composed of CSA, DDB1, DDA1, Cullin 4, and RBX1), STK19, and UVSSA. The transcription elongation factor ELOF1 facilitates the positioning of CRL4^CSA^ and UVSSA within the complex. The central unstructured segment of UVSSA enables the recruitment of TFIIH by interacting with the pleckstrin homology domain of its p62 subunit. STK19 is depicted as a critical factor that interacts with CSA, UVSSA, and the RNAPII subunit RPB1, and accommdates downstream DNA. Through its interaction with the XPD subunit, STK19 positions TFIIH at the DNA region downstream of the lesion-stalled RNAPII, enhancing damage verification and facilitating downstream TCR activities. The figure was created with Biorender.com.

All four landmark studies highlight STK19 as a critical factor in TCR, bridging initial RNAPII stalling and damage recognition to TFIIH recruitment and positioning (Fig. 1). They collectively demonstrate that STK19 interacts with multiple TCR components, including CSA, UVSSA, and RPB1 within the TCR complex. Furthermore, STK19’s role in positioning TFIIH emerges as a common theme, with strong implications for its contribution to damage verification and subsequent repair steps. While previous research established that TFIIH can be initially recruited to the TCR complex through RPB1 ubiquitination (Geijer et al., 2021; van der Weegen et al., 2021) and an interaction between the central unstructured segment of UVSSA and the N-terminal pleckstrin homology domain of p62, a TFIIH subunit (Okuda et al., 2017), these recent studies highlight STK19’s critical role in accurately aligning TFIIH for damage verification and facilitating downstream TCR activities (Fig. 1).

While these studies agree on STK19’s essential role in coordinating TCR, they report differing impacts of STK19 on RPB1 ubiquitination. Ramadhin et al. demonstrated a requirement for STK19 in RPB1 ubiquitination, whereas Tan et al. and van den Heuvel et al. observed little to no impact of STK19 on this modification. Additionally, van den Heuvel et al. demonstrated mild effects of STK19 on TFIIH recruitment and UVSSA ubiquitination, whereas Ramadhin et al. showed a dramatic effect of STK19 on TFIIH recruitment, and Tan et al. highlighted a significant impact on UVSSA ubiquitination. Moreover, how STK19 influences the assembly of the TCR complex, particularly in recruiting UVSSA and TFIIH, varies across studies. Ramadhin et al. found that STK19 is essential for recruiting and stabilizing both UVSSA and TFIIH. In contrast, Tan et al. reported that while STK19 is not required for UVSSA recruitment, it is necessary for UVSSA mono-ubiquitination and TFIIH recruitment. van den Heuvel et al. concluded that STK19 is not essential for recruiting UVSSA or TFIIH, with only a moderate effect on UVSSA mono-ubiquitination. These discrepancies may arise from differences in experimental systems, methods, or biological contexts and warrant further investigation.

Collectively, these recent studies lay a foundation for a more detailed exploration of the intricate mechanisms underlying TCR. Key questions remain unanswered: How does STK19 regulate the ubiquitination of RPB1 and UVSSA, and how do these modifications contribute to the recruitment and activity of TFIIH? Which specific repair factors drive the dissociation of stalled RNAPII? Notably, while the AlphaFold-predicted interactions between STK19 and XPD provide a compelling model, the structure of the STK19-containing TCR complex in conjunction with TFIIH has yet to be resolved. Addressing these questions will be crucial to fully elucidate the role of STK19 in coordinating TCR activities.
